# High serum klotho levels are inversely associated with the risk of low muscle mass in middle-aged adults: results from a cross-sectional study

**DOI:** 10.3389/fnut.2024.1390517

**Published:** 2024-05-24

**Authors:** Yilian Xie, Kai Huang, Hui Li, Weiliang Kong, Jiayuan Ye

**Affiliations:** ^1^Department of Infectious Diseases, The First Affiliated Hospital of Ningbo University, Ningbo, Zhejiang, China; ^2^Department of Hepatology, The First Affiliated Hospital of Ningbo University, Ningbo, Zhejiang, China; ^3^Department of General Medicine, The First Affiliated Hospital of Ningbo University, Ningbo, Zhejiang, China; ^4^Health Science Center, Ningbo University, Ningbo, Zhejiang, China; ^5^Department of Respiratory and Critical Care Medicine, The First Affiliated Hospital of Ningbo University, Ningbo, Zhejiang, China; ^6^Department of Infectious Diseases, Shangyu People's Hospital of Shaoxing, Shaoxing, Zhejiang, China

**Keywords:** klotho, appendicular muscle mass, low muscle mass, NHANES, cross-sectional

## Abstract

**Objective:**

Muscle mass gradually declines with advancing age, and as an anti-aging protein, klotho may be associated with muscle mass. This study aims to explore the relationship between klotho levels and muscle mass in the middle-aged population.

**Methods:**

Utilizing data from the National Health and Nutrition Examination Survey (NHANES) spanning 2011 to 2018, we conducted a cross-sectional analysis on a cohort of individuals aged 40–59. Weighted multivariable analysis was employed to assess the correlation between klotho and low muscle mass, with stratified and Restricted Cubic Spline (RCS) analyses.

**Results:**

The cross-sectional investigation revealed a significant negative correlation between klotho levels and the risk of low muscle mass (Model 3: OR = 0.807, 95% CI: 0.712–0.915). A notable interaction between klotho and sex was observed, with a significant interaction effect (P for interaction = 0.01). The risk association was notably higher in females. The risk association was notably higher in females. Additionally, RCS analysis unveiled a significant linear relationship between klotho and low muscle mass (P for nonlinear = 0.9495, P for overall<0.0001).

**Conclusion:**

Our observational analysis revealed a noteworthy inverse relationship between klotho and low muscle mass, particularly prominent among female participants. This discovery provides crucial insights for the development of more effective intervention strategies and offers a new direction for enhancing muscle quality in the middle-aged population.

## 1 Introduction

Population aging is a global phenomenon with significant implications for healthcare and society. In 2018, the number of people aged 65 and older surpassed the number of children under 5 worldwide for the first time in history (1). By 2050, the population of people aged 80 and older is expected to triple (1). As life expectancy increases, the demand for strategies that promote not just longer life but also longer healthy life grows. The primary obstacle to achieving this goal is frailty, with sarcopenia being a core component and considered as strong predictors of morbidity, disability, and death in older people (2).

Sarcopenia is a syndrome characterized by reduced muscle mass, weakened muscle strength, and decreased functional performance, closely associated with the aging process (3–5). Sarcopenia is correlated with impaired bodily functions, disability, increased rates of falls, and mortality (6). The etiology of sarcopenia is multifaceted, potentially involving factors such as muscle disuse, changes in endocrine function, chronic diseases, inflammation, insulin resistance, and nutritional deficiencies (3). The description of sarcopenia remains a controversial topic, with its definition predominantly focusing on the presence of skeletal muscle atrophy, commonly referred to as low muscle mass (7). Low muscle mass is influenced by various factors, including aging, genetic predisposition, levels of physical activity, dietary habits, and diseases (8, 9). Studies indicate that muscle mass and physical function decline with age, even in highly active older individuals, as muscle mass and strength remain significantly lower than in younger counterparts, highlighting the close relationship between low muscle mass and aging (10–12).

Klotho is a beta-glucuronidase associated with the mechanisms of aging (13). The primary changes observed in body composition during the aging process include a decrease in lean body mass, bone mineral density, and muscle mass, along with an increase in fat body mass (14). Animal-based experiments suggest that elevating klotho levels may enhance skeletal muscle strength, quality, and post-injury recovery, pointing towards a possible key role for klotho in addressing age-related declines in muscle mass (15–17).

While the risk of developing muscle loss increases with age, maintaining optimal muscle health in middle age is crucial for late-life muscle conditions. Investigating the relationship between klotho protein and declining muscle mass in middle-aged individuals provides essential insights for preventive strategies. Therefore, utilizing data from the National Health and Nutrition Examination Survey (NHANES), which represents the entire U.S. population, we assessed the association between serum klotho levels and low muscle mass in the middle-aged population (40–59 years).

## 2 Materials and methods

### 2.1 Study design

**Figure 1:**
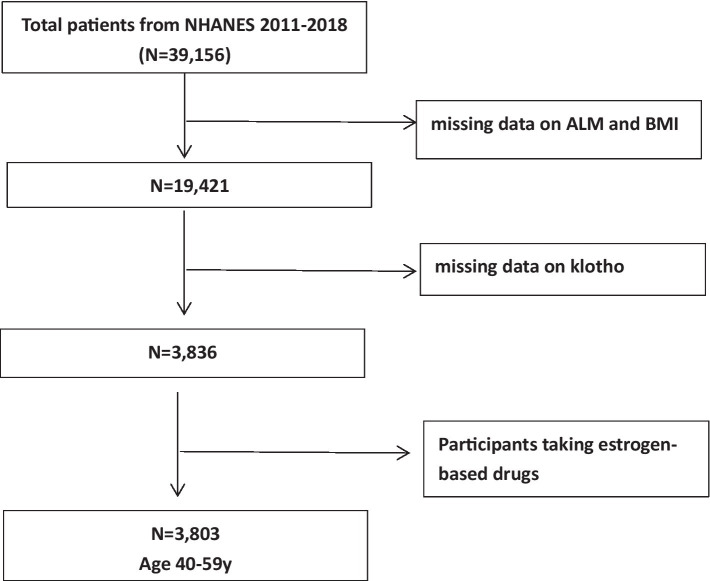
Flow-chart of the study samples.

We conducted a meticulous analysis of data using information derived from the NHANES 2011–2018 cycles, a comprehensive cross-sectional study designed for the American population. For each cycle, a sophisticated, stratified, multistage probabilistic cluster sampling approach was meticulously implemented to ensure the acquisition of samples that accurately represent the entire nation (18). The NHANES survey protocols received ethical approval from the National Center for Health Statistics (NCHS) Research Ethics Review Board, and comprehensive written consent was obtained from all participants. A total of 39,156 individuals actively participated in the NHANES 2011–2018 cycles. We excluded participants lacking data on ALM, body mass index (BMI), and serum α-klotho concentrations. Subsequently, we reviewed the medications used by the participants and excluded those taking estrogen-based drugs, as these could potentially affect klotho levels. Finally, we retained a cohort of 3,803 middle-aged individuals, aged 40 to 59 years, as our study population (Figure 1).

### 2.2 Measurement of serum α-klotho

Serum samples were maintained at −80°C until analysis, which was conducted using a commercially available enzyme-linked immunosorbent assay (ELISA) kit provided by IBL International in T Japan. Two separate analyses were carried out for each sample, and the final result was determined by calculating the average of the two values. The analysis of α-klotho concentrations was similarly conducted in duplicate on each ELISA plate, adhering to rigorous quality control procedures.

### 2.3 Low muscle mass

Height (m) and weight (kg) measurements were conducted for each survey cycle carried out from 2011 to 2018. BMI was derived by dividing weight by the square of height. DXA was employed to assess the ALM for all eligible participants. ALM, considered a reliable proxy for skeletal muscle mass, was calculated by summing the lean mass (excluding bone mineral content) of the right and left leg, as well as the right and left arm, as measured by DXA. Recognized as a pivotal element in sarcopenia, low muscle mass was defined based on the guidelines provided by the Foundation for the National Institutes of Health (FNIH). Specifically, low muscle mass was determined when the ALM/BMI < 0.512 for women and < 0.789 for men.

### 2.4 Definition of covariates

In our analysis, we considered several potential influencing factors as covariates: sex (female, male), age, race (Non-Hispanic white, Non-Hispanic black, Mexican American, others), education level (less than 9th grade, 9–11th grade, high school graduate, some college or AA degree, college graduate or above), smoking habits (nonsmokers, former smokers, and current smokers), alcohol use (nondrinker, mild, moderate, and heavy drinkers) (19), hypertension status (yes, no) (20), type 2 diabetes status (T2DM) (yes, no) (21), physical activity [low, moderate, high, or very high, based on Metabolic equivalents (MET-minutes/week)] (22). Additionally, we included measurements such as estimated glomerular filtration rate (eGFR) (calculated using the Chronic Kidney Disease Epidemiology Collaboration equation (23)), urea nitrogen, proteinuria, serum 25-hydroxyvitamin D, serum calcium, serum phosphorus, and lumbar spine bone mineral density as relevant covariates in our analysis. Chronic kidney disease (CKD) was defined as an eGFR below 60 mL/min/1.73m^2^ or a urinary albumin-to-creatinine ratio of 30 mg/g or higher (23). The serum creatinine and urinary albumin and creatinine values were based on a single measurement from NHANES data (24). Total energy intake (kcal) and protein intake (g), averaged over a two-day period, were also considered in our analysis as important covariates.

### 2.5 Statistical analysis

Categorical variables were presented as weighted proportions ± Standard Error (SE), while continuous variables were represented as weighted means ± SE within the dataset. Linear regression was subsequently performed for continuous variables, and the Rao-Scott chi-square test was applied for intergroup comparisons of categorical variables. Multiple regression models were then employed to calculate the adjusted odds ratio (OR) for low muscle mass across klotho tertiles, along with estimating the adjusted differences in ALM/BMI across klotho tertiles. The multivariate analysis comprised three models: model 1, unadjusted; model 2, adjusted for age, and race; and model 3, adjusted for age, race, education, alcohol use, smoking status, T2DM status, hypertension status, physical activity, eGFR mL/(min*1.73 m2), urea nitrogen (mg/dl), proteinuria (mg/l), 25-hydroxyvitamin D (nmol/L), calcium (mg/dl), phosphorus (mg/dl), lumbar spine bone mineral density (g/cm2), energy (kcal), and protein (g). ALM/BMI was additionally adjusted for sex in model 2 and model 3. Subgroup analysis was conducted by stratifying the dataset based on age, sex, race, physical activity, T2DM status, and hypertension status. Lastly, we employed restricted cubic spline (RCS) to analyze the potential nonlinear association between serum klotho levels and low muscle mass, with three knots placed at the 10th, 50th, and 90th percentiles, using the median of klotho levels as reference values. Weighted analysis was performed using R software (version 4.3.0) to ensure national representativeness, with statistical significance set at *p* < 0.05.

## 3 Result

### 3.1 Basic characteristic of study participants

In Table 1, we present a weighted characterization of study participants, focusing on the presence of low muscle mass. Among middle-aged American adults, the weighted prevalence of low muscle mass (FNIH) was determined to be 16.79%. Distinct patterns emerged between the two groups. Individuals with low muscle mass were typically older, of Hispanic ethnicity, and had a higher prevalence of hypertension, diabetes, and CKD. Additionally, lower levels of physical activity and education were associated with low muscle mass, while these individuals were less likely to be regular consumers of alcoholic beverages. Furthermore, those with low muscle mass often exhibited higher eGFR and triglyceride levels, with lower creatinine, albumin, 25-hydroxyvitamin D, and lumbar spine bone mineral density. No significant differences were observed with respect to sex, smoking status, urea nitrogen, albumin urine, serum cholesterol, serum total protein, serum calcium, serum phosphorus or the intake of energy and protein.

**Table 1:** Characteristics of the study participants by normal and low muscle mass status defined by FNIH among U.S adults aged 40–59 years.

Variable	Total (*n* = 3,803)	Normal (*n* = 3,120)	Low (*n* = 683)	*p* value
Age	49.68 (0.14)	49.44 (0.15)	50.82 (0.26)	<0.001
Sex				0.94
Male	49.07 (0.03)	49.11 (1.13)	48.89 (2.31)	
Female	50.93 (0.03)	50.89 (1.13)	51.11 (2.31)	
Race				<0.001
Non-Hispanic White	69.47 (0.05)	70.17 (2.43)	66.02 (2.76)	
Non-Hispanic Black	9.51 (0.01)	9.91 (1.13)	7.49 (1.20)	
Hispanic	7.92 (0.01)	6.84 (0.97)	13.27 (1.96)	
Other Race	13.10 (0.01)	13.08 (1.14)	13.21 (1.59)	
Smoke status				0.2
Now	21.26 (0.01)	21.81 (1.23)	18.53 (1.64)	
Former	24.20 (0.02)	23.48 (1.10)	27.77 (2.63)	
Never	54.53 (0.03)	54.69 (1.52)	53.71 (2.45)	
Alcohol user				<0.001
Non	8.77 (0.01)	7.84 (0.77)	13.37 (1.81)	
Mild	33.70 (0.02)	34.75 (1.37)	28.52 (2.92)	
Moderate	17.64 (0.02)	18.34 (1.01)	14.20 (1.94)	
Heavy	21.32 (0.01)	21.46 (1.24)	20.63 (2.13)	
Hypertension	18.57 (0.01)	17.61 (0.90)	23.29 (1.83)	
No				<0.001
Yes	60.48 (0.04)	62.21 (1.01)	51.92 (2.54)	
T2DM	39.52 (0.02)	37.79 (1.01)	48.08 (2.54)	
No				<0.001
Yes	85.84 (0.05)	87.72 (0.87)	76.51 (1.92)	
CKD	14.16 (0.01)	12.28 (0.87)	23.49 (1.92)	
No				<0.001
Yes	89.82 (0.05)	90.98 (0.64)	84.94 (1.88)	
Physical activity	10.02 (0.01)	9.02 (0.64)	15.06 (1.88)	
Low				<0.001
Moderate	13.20 (0.01)	13.23 (0.81)	13.03 (1.61)	
High	10.18 (0.01)	10.28 (0.79)	9.70 (2.02)	
Very high	7.81 (0.01)	8.37 (0.69)	5.04 (1.07)	
Education	46.95 (0.03)	48.34 (1.22)	40.02 (2.41)	
Less than 9th grade				<0.001
9–11th grade	4.58 (0.00)	3.88 (0.43)	8.07 (1.22)	
High school graduate	9.33 (0.01)	8.76 (0.99)	12.14 (1.47)	
Some college or AA degree	20.55 (0.02)	20.72 (1.27)	19.71 (2.02)	
College graduate or above	31.41 (0.02)	31.35 (1.31)	31.71 (2.32)	
Protein (g)	84.55 (0.74)	85.32 (0.87)	80.72 (2.42)	0.1
Energy (kcal)	2201.11 (18.56)	2223.05 (21.43)	2092.20 (58.68)	0.06
Klotho (pg/ml)	0.76 (0.01)	0.81 (0.00)	0.51 (0.01)	<0.0001
ALM/BMI	29.73 (0.18)	28.87 (0.15)	34.02 (0.54)	<0.0001
BMI (kg/m^2^)	92.81 (0.46)	92.48 (0.47)	94.42 (0.92)	0.04
eGFR mL/(min*1.73 m^2^)	0.87 (0.01)	0.87 (0.01)	0.84 (0.01)	0.01
Creatinine (mg/dl)	13.34 (0.13)	13.25 (0.14)	13.80 (0.31)	0.09
Urea nitrogen (mg/dl)	27.20 (2.85)	23.86 (2.72)	43.69 (9.52)	0.05
Albumin urine	135.64 (3.91)	130.91 (4.00)	155.76 (9.15)	0.01
Serum triglyceride (mg/dl)	203.38 (1.11)	203.25 (1.15)	204.04 (2.09)	0.71
Serum cholesterol (mg/dl)	43.07 (0.10)	43.20 (0.09)	42.38 (0.18)	<0.0001
Serum albumin (g/l)	64.06 (0.35)	64.06 (0.31)	64.04 (1.28)	0.99
Serum total protein (g/l)	975.52 (12.99)	981.32 (14.77)	946.74 (38.35)	0.43
Serum calcium (mg/dl)	1432.50 (13.19)	1446.46 (15.71)	1363.19 (48.23)	0.14
Serum phosphorus (mg/dl)	71.13 (1.12)	71.74 (1.15)	68.13 (1.63)	0.02
Serum 25-hydroxyvitamin D (nmol/L)	1.02 (0.00)	1.03 (0.00)	1.00 (0.01)	0.01
Lumbar spine bone mineral density (g/cm^2^)	84.55 (0.74)	85.32 (0.87)	80.72 (2.42)	0.1

Data are expressed as weighted proportions ± Standard Error (SE) for categorical variables and as weighted means ± SE for continuous variables. Linear regression and Rao-Scott chi-square test were used to compare groups.ALM, appendicular lean mass; BMI, body mass index; T2DM, type 2 diabetes status; CKD, chronic kidney disease; eGFR, estimated glomerular filtration rate.

### 3.2 Association between klotho and low muscle mass

Conducting a multivariate regression analysis (Table 2), we explored the relationship between klotho and low muscle mass. In the fully adjusted Model 3, our results demonstrated a significant negative correlation between klotho levels and the risk of low muscle mass (Model 3: OR = 0.807, 95% CI: 0.712–0.915). When categorizing klotho from a continuous to a tertile variable, participants in tertiles 3 exhibited a 31.8, 31.9, and 43.1% reduced risk of low muscle mass, respectively, compared to tertiles 1 in all three models. A significant linear trend emerged for the correlation between klotho tertiles and low muscle mass status (Model 1: *p* = 0.032; Model 2: *p* = 0.030; Model 3: *p* = 0.001). These findings suggest that individuals with elevated klotho levels are less susceptible to developing low muscle mass.

**Table 2:** Weighted multivariable odds ratio (OR) for low muscle mass status defined by FNIH based on klotho.

	Model 1 OR (95% CI)	*p*	Model 2 OR (95% CI)	*p*	Model 3 OR (95% CI)	*p*
klotho (per SD increase)	0.891 (0.775, 1.024)	0.102	0.893 (0.779, 1.024)	0.103	0.807 (0.712, 0.915)	0.002
Q1 (151.3–721.8)	Reference		Reference		Reference	
Q2 (721.9–932.9)	0.865 (0.638, 1.171)	0.339	0.850 (0.626, 1.153)	0.287	0.826 (0.601, 1.136)	0.229
Q3 (932.9–5038.3)	0.682 (0.482, 0.965)	0.031	0.681 (0.483, 0.961)	0.030	0.569 (0.414, 0.783)	0.001
P for trend		0.032		0.03		0.001

Model 1: unadjusted covariates; Model 2: adjusted for age, and race; Model 3: adjusted for age, race, education, alcohol use, smoking status, T2DM, type 2 diabetes status. Status, hypertension status, physical activity, eGFR, urea nitrogen, proteinuria, serum 25-hydroxyvitamin D, serum calcium, serum phosphorus, lumbar spine bone mineral density, energy(kcal), and protein(g). FNIH, Foundation for the National Institutes of Health; T2DM, type 2 diabetes status; eGFR, estimated glomerular filtration rate.

In Table 3, we present the relationships between klotho and ALM/BMI. Model 3 indicated a significant positive correlation between serum klotho concentrations and ALM/BMI. Notably, the highest klotho tertile displayed a more pronounced positive association with ALM/BMI compared to the lowest klotho tertile (Model 3: β = 0.019, 95% CI: 0.004–0.035).

**Table 3:** Weighted multivariable associations between klotho and ALM/BMI.

	Model 1 β (95% CI)	*p*	Model 2 β (95% CI)	*p*	Model 3 β (95% CI)	*p*
klotho (per SD increase)	−0.005 (−0.013, 0.003)	0.213	0.006 (0.000, 0.011)	0.060	0.009 (0.004, 0.014)	0.001
Q1(151.3–721.8)	Reference		Reference		Reference	
Q2(721.9–932.9)	0.009 (−0.014, 0.032)	0.446	0.008 (−0.007, 0.024)	0.273	0.008 (−0.006, 0.023)	0.255
Q3(932.9–5038.3)	−0.004 (−0.027, 0.019)	0.711	0.016 (0.000, 0.032)	0.056	0.019 (0.004, 0.035)	0.017
P for trend		0.727		0.055		0.016

Model 1: unadjusted covariates; Model 2: adjusted for sex, age, and race; Model 3: adjusted for age, sex, race, education, alcohol use, smoking status, T2DM status, hypertension status, physical activity, eGFR, urea nitrogen, proteinuria, serum 25-hydroxyvitamin D, serum calcium, serum phosphorus, lumbar spine bone mineral density, energy(kcal), and protein(g). ALM, appendicular lean mass; BMI, body mass index; T2DM, type 2 diabetes status; eGFR, estimated glomerular filtration rate.

### 3.3 Subgroup analysis

**Figure 2:**
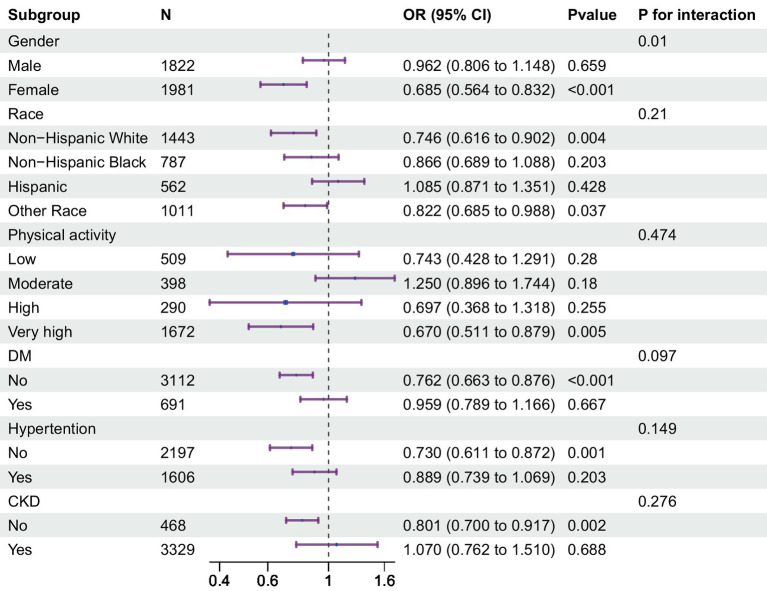
Multivariable odds ratio (or) for low muscle mass status based on klotho stratified by sex, age, race, T2DM and hypertension. Each stratification adjusted for all the factors (age, race, education, alcohol use, smoking status, T2DM status, hypertension status, physical activity, eGFR, urea nitrogen, proteinuria, serum 25-hydroxyvitamin D, serum calcium, serum phosphorus, lumbar spine bone mineral density, energy(kcal), and protein(g)) except the stratification factor itself. T2DM, type 2 diabetes status; eGFR, estimated glomerular filtration rate.

To further elucidate the intricate connection between klotho and low muscle mass status, we conducted subgroup analyses and interaction tests within predefined subgroups (Figure 2). Within these subgroups, a notable interaction between klotho and sex was observed, with a significant interaction effect (P for interaction = 0.01). The risk association was notably higher in females. Conversely, the interaction tests for the other subgroups did not yield significance (P for interaction >0.05). This implies that the sex group plays a substantial role in moderating the relationship between klotho and low muscle mass.

### 3.4 Dose-response relationships between klotho and low muscle mass

**Figure 3:**
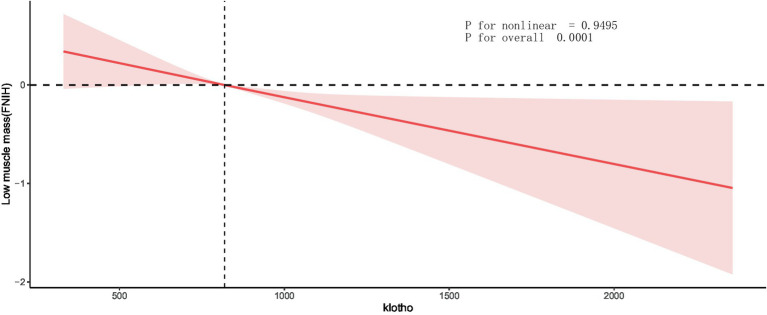
Dose-response relationship between klotho and low muscle mass. Values represent difference in predicted response in reference to a klotho of mean. Red solid lines represent restricted cubic spline models. Adjusted for age, race, education, alcohol use, smoking status, T2DM status, hypertension status, physical activity, eGFR, urea nitrogen, proteinuria, serum 25-hydroxyvitamin D, serum calcium, serum phosphorus, lumbar spine bone mineral density, energy(kcal), and protein(g). ALM, appendicular lean mass; BMI, body mass index; T2DM, type 2 diabetes status; eGFR, estimated glomerular filtration rate.

We also assessed the dose-response relationship between klotho and the prevalence of low muscle mass. Figure 3 visually depicts the dose-response relationship between klotho and low muscle mass. Our analysis indicates a significant linear correlation between klotho and low muscle mass (P for nonlinear = 0.9495, P for overall <0.0001). This suggests that as serum klotho levels increase, the risk of low muscle mass decreases.

## 4 Discussion

This study conducted a comprehensive assessment of the relationship between klotho and muscle mass for the first time. The research integrated cross-sectional NHANES data from 2011 to 2018 to elucidate the association between klotho levels and muscle mass. Our observational analysis, for the first time, reported a significant negative correlation between klotho levels and low muscle mass in middle-aged individuals, particularly pronounced in female participants. Dose-response curves indicated a linear relationship between klotho levels and low muscle mass.

Currently, there are several research reports on the relationship between klotho levels and grip strength. In a prospective study, Semba et al. found that older adults (71–80 years) with higher plasma klotho concentrations experienced less decline in knee strength over 4 years compared to those with lower klotho levels, suggesting that lower plasma klotho concentration predicts changes in skeletal muscle strength (25). Another prospective cohort study in Italian adults aged ≥55 revealed a positive correlation between plasma klotho concentration and lower limb physical function (26). Additionally, a longitudinal aging study in Italians aged 65 and above found that older adults with lower plasma klotho had weaker skeletal muscle strength (27). However, there is currently a very limited number of clinical studies investigating the relationship between klotho and muscle mass. Only one small-sample study with 74 participants has been identified, suggesting a strong positive correlation between lean mass index and S-klotho plasma levels in middle-aged individuals (28). Research indicates that muscle mass gradually decreases with age, with adults losing ~20% of their skeletal muscle mass between 40 and 80 years old (29, 30). Studies on klotho show a significant decline after the age of 40 (14, 31). As an anti-aging protein, klotho’s most significant role is in slowing the aging and disuse of various organs, including muscles (32), suggesting a possible connection between the two. Our study found a negative correlation between klotho and low muscle mass in the middle-aged population (40–59 years), indicating that higher klotho levels are associated with a lower risk of low muscle mass. This provides important clues for understanding the role of klotho in middle-aged muscle health and valuable references for future interventions and clinical research.

Interestingly, in subgroup analysis, we observed gender differences in the relationship between klotho levels and low muscle mass, with klotho exhibiting a more pronounced protective effect against low muscle mass risk in females. Currently, there are no similar studies reported in the literature. However, animal experiments have shown that overexpression of the klotho gene can enhance voluntary running capacity and improve muscle function in mice with Duchenne muscular dystrophy, a condition characterized by muscle degeneration, with female mice showing more significant improvements (33). This finding suggests that further exploration of the gender-specific effects of klotho on muscle health in human populations may yield valuable insights into potential therapeutic strategies and preventive measures against age-related muscle decline.

The progression of aging in adults is closely linked to significant alterations in body composition, characterized primarily by a reduction in skeletal muscle mass (34). Currently, the mechanisms underlying muscle loss remain poorly understood, and treatment strategies primarily focus on physical activity and dietary adjustments. Additionally, advanced clinical trials are exploring therapeutic avenues involving vitamin D, insulin-like growth factor, testosterone, and monoclonal antibodies (15). Klotho, with its anti-aging properties, plays a crucial role in regulating fibroblast growth factor 23 (FGF23), Wnt, and mTOR pathways, several of which are targets in the study of muscle loss (35–38). These targets include the regulation of mitochondrial reactive oxygen species, contributing to decreased muscle mass and regenerative potential (16, 39), modulation of extracellular Wnt binding to reduce Wnt signaling, enhancing muscle growth and regeneration post-injury (17, 40–42), and involvement in reducing oxidative stress (43). Additionally, klotho acts through the activation of downstream targets required for protein synthesis, playing a role in transforming growth factor-β1 and insulin/insulin-like growth factor-I signaling by activating PI3K/Akt pathways, thereby contributing to skeletal muscle hypertrophy (44–46). Klotho further reduces inflammation in endothelial cells by attenuating nuclear factor-κ B activation and enhancing tumor necrosis factor-α-induced adhesion molecule expression (47). Therefore, klotho may represent a novel avenue for preventing or reversing muscle loss.

Current therapeutic approaches to enhance klotho expression include the administration of recombinant klotho protein (48). Additionally, strategies involving the supplementation of potential nutrients that boost endogenous klotho production, such as vitamin D (49), and increased dietary fiber intake (50) have been explored. Studies by Lu et al. suggested that maintaining carbohydrate intake between 48.92 and 56.20% is associated with the highest serum klotho levels (51). Amaro-Gahete et al.’s research indicated that exercise training can elevate S-klotho plasma levels in sedentary middle-aged individuals (52). Collectively, through interventions like exercise and dietary adjustments, we have the potential to modulate klotho levels, influencing muscle health. This discovery provides crucial insights for the development of more effective intervention strategies and offers a new direction for enhancing muscle quality in the middle-aged population.

The main strength of this study lies in the first exploration of the relationship between klotho and the risk of low muscle mass using data from the large-scale cross-sectional NHANES study. However, the study has some potential limitations. Firstly, due to the limitations of the nature of cross-sectional studies, it was difficult to establish causality. Higher-level research, such as prospective studies, is needed. Secondly, some self-reported confounders may be subject to biased recall. Finally, there is a possibility of missing some potentially confounding variables, such as FGF23, Parathyroid hormone, which could introduce bias.

## 5 Conclusion

Our cross-sectional study suggests that elevated klotho levels are associated with a reduced risk of muscle loss, with a more pronounced effect observed in females. These findings preliminarily reveal a potential association between klotho and muscle loss. Therefore, elevating klotho levels could potentially be developed as a novel approach to prevent or reverse muscle loss.

## Data availability statement

Publicly available datasets were analyzed in this study. This data can be found here: https://www.cdc.gov/nchs/nhanes/.

## Ethics statement

The studies involving humans were approved by the National Center for Health Statistics (NCHS) Research Ethics Review Board. The studies were conducted in accordance with the local legislation and institutional requirements. The participants provided their written informed consent to participate in this study.

## Author contributions

YX: Writing – original draft, Writing – review & editing, Conceptualization, Funding acquisition, Investigation, Methodology. KH: Data curation, Writing – original draft. HL: Data curation, Writing – original draft. WK: Investigation, Methodology, Software, Writing – original draft, Writing – review & editing. JY: Conceptualization, Supervision, Writing – review & editing.
